# Supplementary material for: Integrative Metabolome and Proteome Analysis of Cerebrospinal Fluid in Parkinson’s Disease
Source: Int J Mol Sci. 2024 Oct 23;25(21):11406. doi: 10.3390/ijms252111406 (PMC11547079; doi:10.3390/ijms252111406)
Supplement: Supplementary file 1 [file ijms-25-11406-s001.zip › ijms-3260780-supplementary.pdf]

**Table S1.** List of the top seven inhibited canonical pathways identified through integrative omics analysis.

| No. | Ingenuity canonical pathways | −log(*p*-value) | Activation z-score | Related molecules |
|---|---|---|---|---|
| 1 | Neutrophil degranulation | 2.96 | -2.121 | CO3, CD44, CH3L1, GROA, SAP3, HPT, PTPR2, TTHY |
| 2 | Neurotransmitter clearance | 4.97 | -2.000 | 3,4-dihydroxyphenylacetic acid, 5-hydroxyindol-3-acetic acid, 5-hydroxytryptamine, homovanillic acid |
| 3 | UFMylation signaling | 3.66 | -2.000 | IL1B, IL6, L-glutamic acid, TNF |
| 4 | Sulfur amino acid metabolism | 3.57 | -2.000 | betaine, glutathione disulfide, L-glutamic acid, taurine |
| 5 | Cargo recognition for clathrin-mediated endocytosis | 2.77 | -2.000 | AP2B1, APOB, TGFA, TGON2 |
| 6 | Clathrin-mediated endocytosis | 2.44 | -2.000 | AP2B1, APOB, TGFA, TGON2 |
| 7 | IL-10 signaling | 2.28 | -2.000 | IL1B, IL6, nitric oxide, TNF |

**Table S2.** List of the top seven activated canonical pathways identified through integrative omics analysis.

| No. | Ingenuity canonical pathways | −log(*p*-value) | Activation z-score | Related molecules |
|---|---|---|---|---|
| 1 | Communication between innate and adaptive immune cells | 1.31 | 2.828 | IGHG4, HV102, KV401, LV147, IL1B, IL4, IL6, TNF |
| 2 | IL-33 signaling | 3.77 | 2.449 | CCL2, IL1B, IL4, IL6, nitric oxide, TNF |
| 3 | Activin inhibin signaling | 2.63 | 2.236 | IGHG4, IL1B, IL6, TGFB1, TNF |
| 4 | FCGR dependent phagocytosis | 3.04 | 2.236 | arachidonic acid, IGHG4, HV102, KV401, LV147 |
| 5 | MAPK signaling (inhibiting influenza) | 4.54 | 2.236 | arachidonic acid, CCL2, IL1B, IL6, TNF |
| 6 | NAFLD signaling | 4.99 | 2.121 | APOB, D-fructose, IL17A, IL1B, IL4, IL6, TGFB1, TNF |
| 7 | Dendritic cell maturation | 0.59 | 2.000 | IGHG4, IL1B, IL6, TNF |

**Table S3.** List of the top seven inhibited diseases and biological functions identified through integrative omics analysis.

| No. | Diseases or functions annotation | −log(*p*-value) | Activation z-score | Related molecules |
|---|---|---|---|---|
| 1 | Tubulation of vascular endothelial cells | 8.86 | -2.894 | 5-hydroxytryptamine, APOH, CD44, GROA, SCF, SCG2, PEDF, TGFB1, TNF, VEGFA |
| 2 | Respiration of mitochondria | 7.28 | -2.63 | betaine, inosine, L-glutamic acid, nitric oxide, spermidine, TGFB1, TNF |
| 3 | Mobilization of Ca2+ | 11.91 | -2.579 | 5-hydroxytryptamine, CO3, CO4A/B, CCL2, CCL23, CCL28, CCL8, CD44, corticosterone, GROA, IL16, IL4, SCF, L-glutamic acid, NGF, NPY, TGFB1, TNF, VEGFA |
| 4 | Release of neurotransmitter | 9.15 | -2.578 | 2-phenethylamine, 5-hydroxytryptamine, CO3, corticosterone, IL1B, L-glutamic acid, levodopa, NGF, nitric oxide, NPY, PARK7, TNF |
| 5 | Proliferation of neuronal cells | 9.67 | -2.389 | 5-hydroxytryptamine, APOE, CCL2, CLUS, CSF1, ENPP2, IL17A, IL1B, IL6, inosine, SCF, levodopa, NGF, nitric oxide, NPY, putrescine, TGFA, TGFB1, TNF,VEGFA,VGF |
| 6 | Transmembrane potential of mitochondria | 6.48 | -2.354 | arachidonic acid, IL1B, IL4, IL6, L-glutamic acid, levodopa, N-methyl-(R)-salsolinol, NGF, nitric oxide, PARK7, TGFB1, TNF |
| 7 | Growth of epithelial tissue | 10.97 | -2.894 | 5-hydroxytryptamine, APOH, CD44, GROA, SCF, SCG2, PEDF, TGFB1, TNF, VEGFA |

**Table S4.** List of the top seven activated diseases and biological functions identified through integrative omics analysis.

| No. | Diseases or functions annotation | −log(*p*-value) | Activation z-score | Related molecules |
|---|---|---|---|---|
| 1 | Apoptosis of vascular cells | 8.95 | 3.147 | 5-hydroxytryptamine, arachidonic acid, PD1L1, IL1B, IL6, SCF, RET4, SCG2, taurine, TGFB1, TNF, VEGFA |
| 2 | Apoptosis of vascular endothelial cells | 7.12 | 2.72 | 5-hydroxytryptamine, arachidonic acid, PD1L1, IL1B, RET4, SCG2, TGFB1, TNF, VEGFA |
| 3 | Visceromegaly | 7.50 | 2.586 | 5-hydroxytryptamine, APOE, CO3, CO4A/B, CMGA, corticosterone, CSF1, HPT, IL17A, IL1B, IL4, IL6, nitric oxide, PARK7, NEC1, putrescine, RET4, 7B2, spermidine, taurine, TGFB1, TNF, VEGFA |
| 4 | Adhesion of connective tissue cells | 7.28 | 2.449 | CCL2, CD44, corticosterone, CSF1, IL1B, IL4, IL6, TGFB1, TNF, VEGFA |
| 5 | Aggregation of phagocytes | 10.08 | 2.384 | CD44, IL1B, IL4, IL6, SCF, KLKB1, TGFB1, TNF |
| 6 | Adhesion of lymphocytes | 7.39 | 2.377 | arachidonic acid, CCL2, CCL28, CD44, IL1B, IL4, IL6, TGFB1, TNF |
| 7 | Abnormal morphology of body cavity | 8.15 | 2.356 | 5-hydroxytryptamine, APOE, CO3, CO4A/B, CCL28, PD1L1, CD44, CMGA, CLUS, corticosterone, CSF1, DNS2A, FGF19, HPT, SIAL, IL17A, IL1B, IL4, IL6, nitric oxide, PARK7, NEC1, PTPR2, putrescine, RET4, 7B2, spermidine, taurine, TGFA, TGFB1, TNF, VEGFA, VGF |

**Table S5.** Ingenuity Pathway Analysis (IPA)-based Parkinson's disease-related microRNAs.

| Symbol | Synonyms | Increased/ decreased | Source | Reference |
|---|---|---|---|---|
| miR-126a-5p | hsa-miR-126, hsa-miR-126-5p, miR-126, miR-126-5p, miR-126a-5p | Decreased | PBMC | [143] |
| miR-151-3p | hsa-miR-151-3p, hsa-miR-151a-3p, miR-151, miR-151-3p, miR-151a-3p | Decreased | PBMC | |
| miR-151-5p | hsa-miR-151-5p, hsa-miR-151a-5p, hsa-miR-151b, miR-151, miR-151-5p, miR-151a-5p, miR-151b | Decreased | PBMC | |
| miR-199a-5p | hsa-miR-199-s, hsa-miR-199a, hsa-mir-199a-1-5p, hsa-mir-199a-2-5p, hsa-miR-199a-5p, hsa-miR-199b-5p, miR-199a-5p, miR-199b, miR-199b-5p, Mir199-5p | Decreased | PBMC | |
| miR-23a-3p | hsa-miR-23a-3p, hsa-miR-23b-3p, hsa-miR-23c, miR-23a, miR-23a-3p, miR-23b-3p, miR-23c | Increased<br>Decreased | Plasma<br>Small extracellular vesicles from plasma | [144] |
| miR-708-5p | hsa-miR-3139, hsa-miR-28-5p, hsa-miR-708-5p, miR-3139, miR-28-5p, miR-708-5p, MIR28A-5p | Decreased | PBMC | [143] |
| mir-126 | HSA-MIR-123, hsa-miR-126, MI0000149, microRNA 123, microRNA 126, microRNA 126a, microRNA 126b, Mir3567, miR-123, Mir126a, Mir126b, MIRN123, MIRN126, miRNA126 | Decreased | PBMC | |
| mir-130 | HSA-MIR-130, hsa-miR-301, hsa-miR-130a, hsa-miR-130b, hsa-miR-301a, hsa-miR-301b, microRNA 130, microRNA 301, microRNA 130a, microRNA 130b, microRNA 301a, microRNA 301b, MIR301, MIR130A, MIR130B, MIR301A, MIR301B, Mirn130, MIRN301, MIRN130A, MIRN130B, MIRN301A, MIRN301B, miRNA130A | Decreased | PBMC | |
| mir-147 | hsa-mir-147, hsa-mir-147a, hsa-miR-147b, microRNA 147, microRNA 147a, microRNA 147b, MIR147A, MIR147B, MIRN147, MIRN147B | Decreased | PBMC | |
| mir-19 | C13orf25, HSA-MIR-19A, hsa-miR-19b, hsa-mir-19b-1, hsa-mir-19b-2, microRNA 19, microRNA 19a, microRNA 19b, microRNA 19b-1, microRNA 19b-2, MIR17HG, MIR19A, MIR19A., miR-19a/b, MIR19B, MIR19B1, MIR19B2, MIRH1, MIRHG1, MIRN19A, Mirn19b, MIRN19B1, MIRN19B2, miRNA19A, miRNA19B1 | Decreased | PBMC | |
| mir-199 | HSA-MIR-199A, hsa-mir-199a-1, hsa-mir-199a-2, Hsa-mir199a-as, hsa-miR-199b, hsa-miR-199-s, MI0000280, microRNA 199, microRNA 199a-1, microRNA 199a-2, microRNA 199b, mir199 3p, mir199 5p, MIR199A, MIR199A1, MIR199A2, miR-199a-3p(2), miR-199a-5p(2), MIR199B, MIR-199-s, Mirn199, Mirn199a, MIRN199A1, MIRN199A2, Mirn199a-as, MIRN199B | Decreased | PBMC | |

| | | | | |
|---|---|---|---|---|
| mir-23 | hsa-miR-23a, hsa-miR-23a/b, hsa-miR-23b, microRNA 23a, microRNA 23b, MIR23A, MIR23B, MIRN23A, MIRN23B, miRNA23A, miRNA23B | Increased | Plasma | [144] |
| mir-26 | HSA-MIR-26A, hsa-miR-26a-1, hsa-mir-26a-2, hsa-miR-26b, MI0000574, microRNA 26a, microRNA 26a-1, microRNA 26a-2, microRNA 26b, MIR26A1, MIR26A2, MIR26A, MIR26B, MIRN26A1, MIRN26A2, MIRN26A, MIRN26B | Decreased | PBMC | [143] |
| mir-28 | hsa-miR-28, hsa-miR-151, hsa-mir-151a, hsa-mir-151b, microRNA 28, microRNA 151, microRNA 151a, microRNA 151b, microRNA 28A, microRNA 28c, MIR151, Mir3586, miR-28-A, MIR151A, MIR151B, Mir28c, MIRN28, MIRN151 | Decreased | PBMC | |
| mir-29 | hsa-miR-102, hsa-mir-29, hsa-miR-29a, HSA-MIR-29B, hsa-mir-29b-1, hsa-mir-29b-2, hsa-mir-29b-3, hsa-miR-29c, MI0000106, microRNA 29, microRNA 29a, microRNA 29b, microRNA 29b-1, microRNA 29b-2, microRNA 29c, microRNA 29 group, microRNA mir-29b-3, microRNA mir-29c-2, miR-102, MIR29A, MIR29B, MIR29B1, MIR29B2, Mir29b-3, MIR29C, Mir29c-2, miR-29 family, MIRN29, MIRN29A, MIRN29B, MIRN29B1, MIRN29B2, MIRN29C, miRNA29A, miRNA29B1, miRNA29C | Decreased | PBMC | |
| mir-30 | hsa-miR-30, hsa-miR-30a, hsa-miR-30a-3p, hsa-miR-30b, HSA-MIR-30BN, HSA-MIR-30C, hsa-mir-30c-1, hsa-mir-30c-2, HSA-MIR-30D, hsa-miR-30e, hsa-miR-30e-5p, hsa-mir-97-6, MI0000099, microRNA 30a, microRNA 30B, microRNA 30C, microRNA 30c-1, microRNA 30c-2, microRNA 30d, microRNA 30e, miR-200, MIR30A, MIR30A3P, MIR30B, MIR30C, MIR30C1, MIR30C2, MIR30D, miR-30d-prec, MIR30E, Mir97, MIRN30A, MIRN30B, Mirn30c, MIRN30C1, MIRN30C2, MIRN30D, MIRN30E, MIRN30E-5P | Decreased | PBMC | |
| mir-335 | hsa-miR-335, microRNA 335, microRNA mir-335, MIRN335, miRNA335 | Decreased | PBMC | |
| mir-374 | hsa-miR-374, hsa-miR-374a, hsa-miR-374b, hsa-miR-374c, microRNA 374, microRNA 374a, microRNA 374b, microRNA 374c, MIR374A, MIR374B, MIR374C, MIRN374, MIRN374A, MIRN374B` | Decreased | PBMC | |

*PBMC: peripheral blood mononuclear cell
